# An overview of common peroneal nerve dysfunction and systematic assessment of its relation to falls

**DOI:** 10.1007/s00264-022-05593-w

**Published:** 2022-09-28

**Authors:** Angelo Capodici, Elisabet Hagert, Halley Darrach, Catherine Curtin

**Affiliations:** 1 Department of Biomedical and Neuromotor Science, Alma Mater Studiorum - University of Bologna, Via San Giacomo 12, 40126 Bologna, Italy; 2 Department of Medicine (Biomedical Informatics), Stanford University - School of Medicine, Stanford, CA USA; 3 Aspetar Orthopedic- and Sports Medicine Hospital, Doha, Qatar; 4 Deparment of Clinical Science and Education, Karolinska Institutet, Stockholm, Sweden; 5 Division of Plastic Surgery, Department of Surgery, Stanford University, Stanford, CA USA; 6 Department of Surgery - Veterans’ Affairs Palo Alto Healthcare System, Palo Alto, CA USA

**Keywords:** Falls, Nerve, Peroneal, Review, Surgery

## Abstract

**Purpose:**

Compression of the peroneal nerve is recognized as a common cause of falls. The superficial course of the peroneal nerve exposes it to trauma and pressure from common activities such as crossing of legs. The nerve can be exposed also to distress due to metabolic problems such as diabetes. The purpose of our manuscript is to review common peroneal nerve dysfunction symptoms and treatment as well as provide a systematic assessment of its relation to falls.

**Methods:**

We pooled the existing literature from PubMed and included studies (*n* = 342) assessing peroneal nerve damage that is related in any way to falls. We excluded any studies reporting non-original data, case reports and non-English studies.

**Results:**

The final systematic assessment included 4 articles. Each population studied had a non-negligible incidence of peroneal neuropathy. Peroneal pathology was found to be consistently associated with falls.

**Conclusion:**

The peroneal nerve is an important nerve whose dysfunction can result in falls. This article reviews the anatomy and care of the peroneal nerve. The literature review highlights the strong association of this nerve’s pathology with falls.

**Supplementary Information:**

The online version contains supplementary material available at 10.1007/s00264-022-05593-w.

## Introduction

The peroneal (or fibular) nerve is a major lower limb nerve. Recently, there has been increased recognition of the association between peroneal nerve dysfunction and fall risk. This paper reviews the common peroneal nerve anatomy, common pathology, symptoms, testing and treatments. This study also pools the literature with a systematic assessment of the peroneal nerve and falls. The aim of this paper is to provide clinicians with a better understanding of this common condition and its relation to falls.

### Anatomy and function

**Fig. 1:**
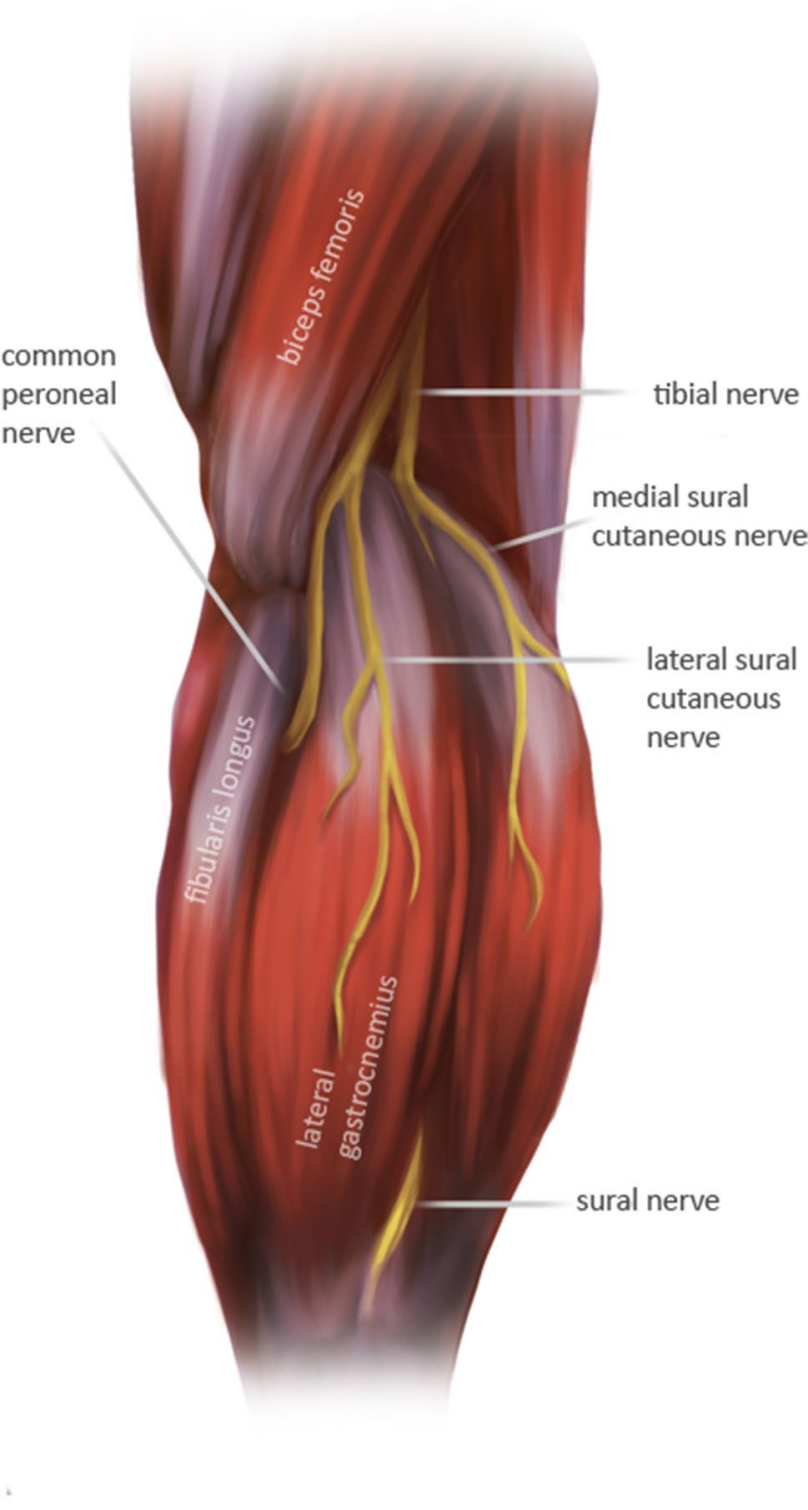
Illustration of the peroneal nerve path, popliteal view, by Halley Darrach

The peroneal nerve originates from the posterior divisions of L4-S2, which then form the sciatic nerve. The sciatic nerve bifurcates into the tibial and peroneal nerves proximal to the popliteal fossa. The common peroneal nerve courses posterolaterally just posterior to the long head of the biceps femoris. The nerve then moves anteriorly wrapping around the fibular neck, 2 cm distal to the fibular head, and passes beneath the lateral compartment on the calf. At or near the fibular neck, the nerve divides into its deep and superficial branches (Fig. 1). The deep peroneal nerve runs within the anterior compartment of the leg between the extensor hallucis longus muscle and the tibialis anterior muscle. It continues down the anterior tibia ultimately terminating between the 1st and 2nd toes. The superficial peroneal runs in the lateral compartment of the leg, before reaching the dorsum of the ankle and foot [1–4].

The peroneal nerve is a sensory-motor nerve. The motor function consists of dorsiflexion of the foot and toe extension through the deep peroneal branch and ankle eversion through the superficial branch. The sensory component of the superficial peroneal nerve supplies the dorsum of the foot except for the webspace between the first and second toes which is supplied by the deep peroneal nerve [1].

### Dysfunction, symptoms, physical and diagnostic testing

The common peroneal nerve is at risk of compression and injury as it wraps around the fibular neck. At this location, the nerve is superficial and is transitioning beneath the lateral compartment muscles resulting in a higher exposure to trauma and swelling. Consequently, common aetiologies for peroneal dysfunction are mechanical-related, such as injuries and surgery around the knee. Mechanical, non-traumatic causes of peroneal nerve compression include too tight bandages, prolonged leg crossing and positioning during anaesthesia [2, 3, 5].

There are also non-mechanical reasons for peroneal dysfunction, such as Charcot-Marie-Tooth disease and polyarteritis nodosa [6], but diabetes is by far the most common. Diabetes is a high prevalence disease impacting 19.3% of adults older than 65. Diabetes is a leading cause of mortality and morbidity especially impacting peripheral nerves such as the peroneal nerve [7, 8]. High blood sugar, reduced blood flow and high triglyceride and cholesterol levels work in synergy to cause peripheral nerve damage. Studies assessing peroneal nerve compression in patients with diabetes show a high incidence ranging from 10 to 60% depending on population and definition [9–14].

Peroneal nerve symptoms range in severity with mild symptoms often unrecognized. Patients may complain of lateral knee pain or frequent tripping. Patients may relate a “drop” foot and tripping accidents catching their foot on uneven surfaces. Sensory symptoms may be numbness on the dorsum of the foot or complaints of a numb, ticklish sensation from the upper lateral calf [15].

The physical examination starts with sensory testing, which can be done by monofilament or Ten-Test sensory examinations in the superficial and deep sensory distributions [16]. Motor testing includes assessing ankle and toe dorsiflexion and ankle eversion. Slight weakness compared to the contralateral side can help localize the pathology. Provocative tests including Tinel and Scratch collapse tests at the fibular neck are also part of the examination [17–19] (Supplementary Material, Video 1).

The diagnostic testing can comprise an electrodiagnostic evaluation, which is more useful in diagnosing a neuropathy with axonal deficits and can help localize a more severe peroneal neuropathy [20]. In milder cases, nerve conduction studies of the peroneal nerve are often normal, limiting the usefulness of electrodiagnostic testing [21]. Imaging, in the form of ultrasound and magnetic resonance neurography, is emerging as an additional tool to identify a swollen peroneal nerve [22]. Ultrasound holds promise as it already has been successfully used to diagnose other entrapment neuropathies such as carpal tunnel syndrome [23].

### Treatments

There are several non-surgical treatments for peroneal nerve compression. A systematic review with meta-analysis [24] found exercise was helpful for patients with diabetic peripheral and chemotherapy-induced neuropathy. Supplements such as vitamin B α-lipoic acid have shown some benefits [25, 26]. Unconventional therapies such as moxibustion were found to be effective in increasing sensory-nerve conduction velocity [27].

**Fig. 2:**
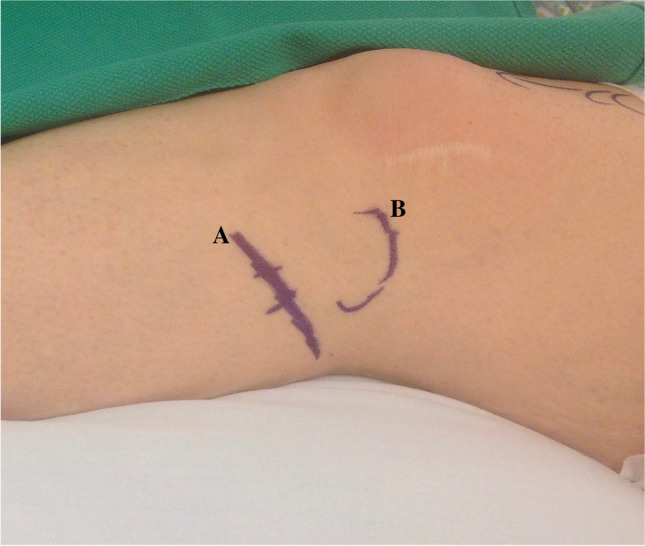
Anatomical region for the incision. Line A underlines the common peroneal nerve course. The fibular head is underlined by line B

**Fig. 3:**
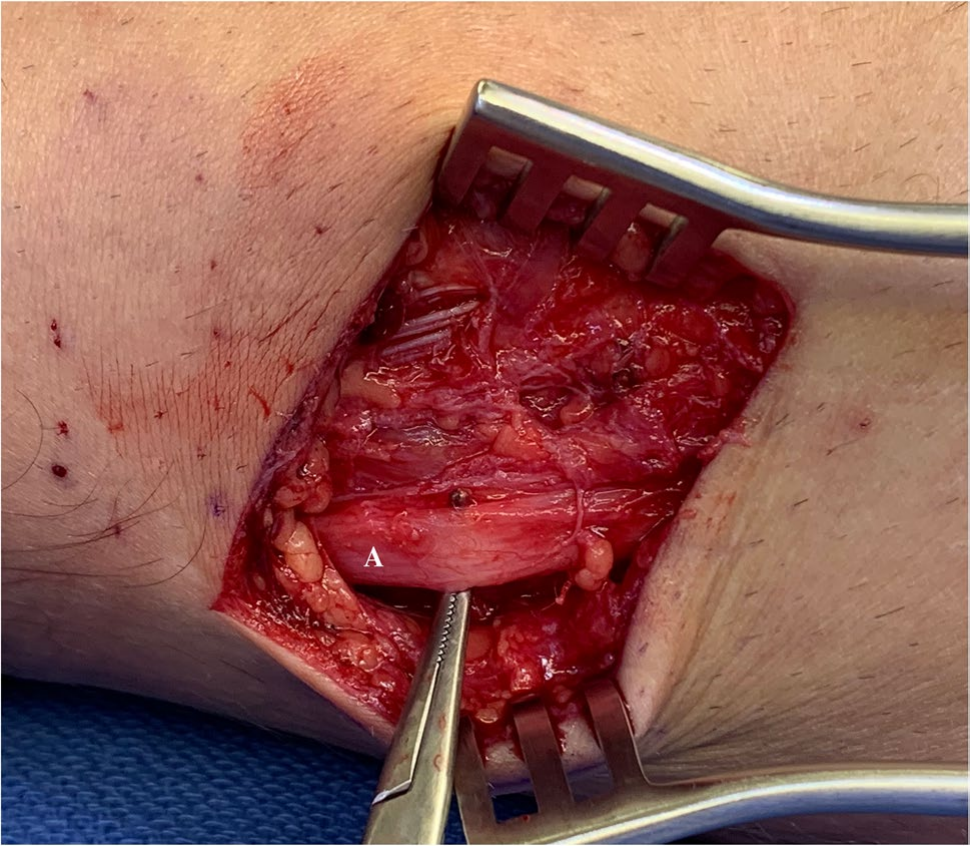
Intraoperative picture of the common peroneal nerve, after the incision. The letter A refers to the common peroneal nerve

Surgical decompression of the common peroneal nerve is a common treatment with increasingly robust evidence of efficacy. A small oblique incision distal to the fibular head provides adequate exposure (Figs. 2 and 3). The proximal nerve is isolated and then neurolysis continues distally including both the deep and superficial branches [28].

A recent meta-analysis found that decompression of the common peroneal nerve is safe and effective with a log(OR) of a favourable outcome after neurolysis of 3.38 (95% *CI*, 2.29–4.48) [29].

## Methods for rapid systematic assessment

A systematic assessment of the common peroneal nerve and its relation to falls was conducted; since this study did not involve contact with patients nor animals, no ethics committee was necessary to assess compliance with Human and Animal Rights. This systematic assessment follows the Preferred Reporting Items for Systematic Reviews (PRISMA) approach [30], including studies that are considered peroneal nerve damage and falls.

The initial PubMed search was implemented on September 1, 2022 and used the following search string: ((perone* OR fibul*) AND nerve* AND (Fall* OR Fell OR Slip* OR Trip* OR Stumbl* OR Collaps*)) AND (("1000/01/01"[Date—Publication]: "2022/09/01"[Date—Publication])) NOT (“congress”[pt] OR “editorial”[pt] OR “meta analysis”[pt] OR “systematic review”[pt] OR review[pt]).

We included articles if they were human-based studies assessing peroneal nerve damage that is related in any way to falls. We excluded studies that did not use English as a language, systematic reviews, opinions, editorials, congress publications, meta-analyses and case reports.

### Data extraction

Data was extracted by one reviewer (AC). Doubt on extracted data was discussed with an independent arbiter (CC). The following data were extracted (main text and/or supplementary material): country of origin, population number, age (median), main pathology, prevalence of peroneal neuropathy and fall association reported.

## Results

**Fig. 4:**
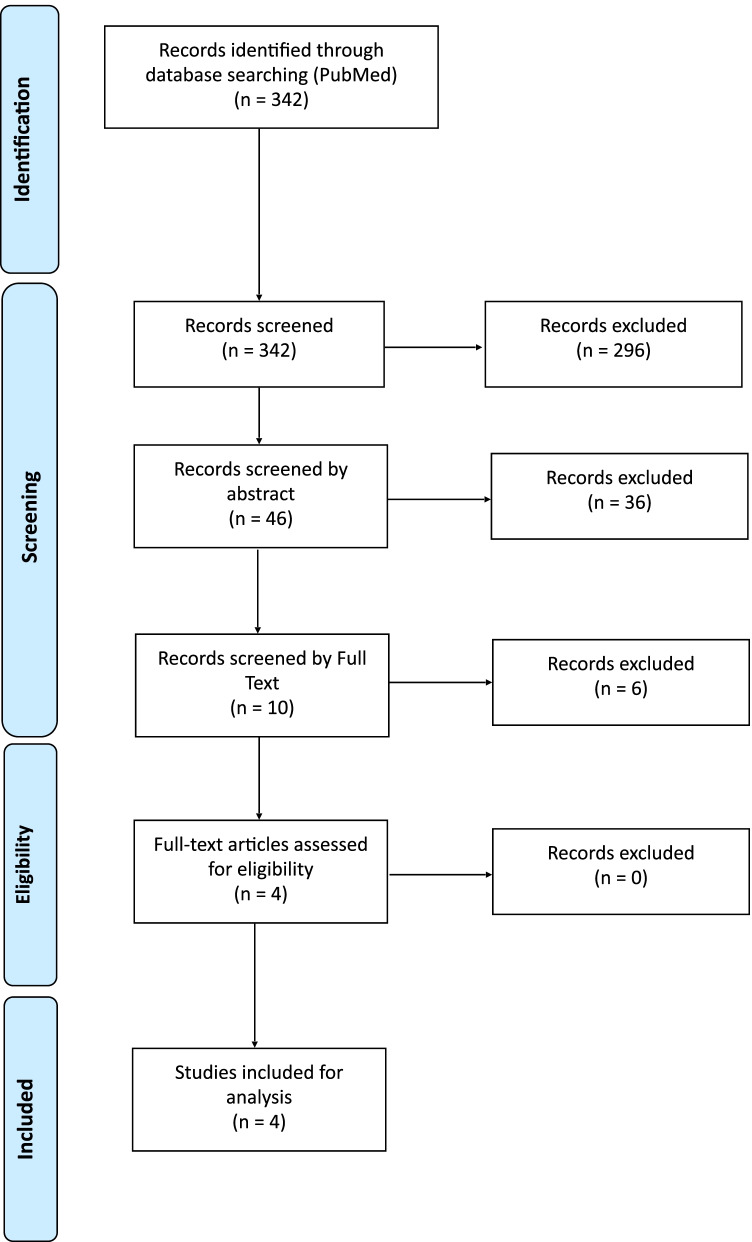
PRISMA flow-chart regarding the selection process

Of the 342 articles retrieved from PubMed, 296 were ruled out after title screening; 36 were excluded after abstract screening; and six were excluded after full-text screening. A total of four articles were selected for data extraction (Fig. 4 and Table 1).

**Table 1:** Studies included into the systematic assessment

Title	1st Author	Year	Country	N. Population	Age, median	Main Pathology	Prevalence of Neuropathy or subclinical neuropathy	Relation to fall described
Subclinical peroneal neuropathy affects ambulatory, community-dwelling adults and is associated with falling	Poppler LH	2020	USA	397	54 ± 15 years	None specified	3.3%, subclinical	Likelihood of self-reporting a fall in the past year increased by 3.7
Subclinical Peroneal Neuropathy: A Common, Unrecognized, and Preventable Finding Associated With a Recent History of Falling in Hospitalized Patients	Poppler LH	2016	USA	100	53 ± 13 years	None specified	67%, subclinical and clinical	Likelihood of self-reporting a fall in the past year increased by 4.7
Diabetes-related complications, glycemic control, and falls in older adults	Schwartz AV	2008	USA	3075	73.6 ± 2.7	Diabetes	22.5%, subclinical	Association between nerve response amplitude and falls: *OR* = 1.71 (*CI* = 1.19–2.44)
Postural stability in diabetic polyneuropathy	Boucher P	1995	Canada	12	62.5 ± 7.4	Diabetes	100%, clinical	Postural instability increased linearly with the severity of the neuropathy (*p* < 0.05)

All the selected studies were published by authors affiliated with institutions based in North America [31–34].

### Demographics

The total enrolled population was 3584 individuals, with a mean age of 60.8 and mean gender distribution of 47.8% females.

The study populations were taken from plastic surgery clinics [31] and general medicine wards [32] and focused on diabetic patients [33, 34].

### Prevalence of peroneal neuropathy

One study [34] assessed equilibrium and stability in diabetic patients having peroneal neuropathy. The following results refer to the remaining 3 studies. One study found a prevalence of 3.3% subclinical peroneal neuropathy in their community-dwelling population [31]. The same first author, in another study, reported a prevalence of 67% of their inpatient sample had at least one sign of subclinical peroneal neuropathy, and 31% of patients had two signs, meeting the definition of subclinical peroneal neuropathy [32]. Finally, a prevalence of 22.5% was found in a diabetic inpatients’ sample, having “loss of light touch discrimination” in the peroneal nerve territory [33].

### Association with falls

Every study reported an association between peroneal nerve dysfunction and falls.

The community dwellers with subclinical peroneal neuropathy were 3.7 times more likely to have a self-reported fall two or more times in the past year [31]. Another study found that patients, who had subclinical peroneal neuropathy, were 4.7 times more likely to have a self-reported fall in the past year, while patients who presented just one sign of subclinical peroneal neuropathy were 2.9 times more likely to self-report a fall [32]. Postural instability, and therefore the possibility of falling, was found to increase linearly with the severity of the neuropathy (*p* < 0.05), heedlessly of vision [34]. Schwartz et al. [33] found an association between decreasing nerve response amplitude in the peroneal nerve with falls with an odds ratio of 1.71 (*CI*: 1.19–2.44).

## Discussion

Common peroneal compression is a common nerve entrapment with incidence similar to other compression neuropathies such as ulnar nerve compression at the elbow. The systematic assessment found a strong association between peroneal nerve neuropathy and falls. Clinicians should have a heightened awareness for this condition in elders, people with diabetes and those complaining of frequent stumbles. The first step to improving outcomes is recognition. Identification of this neuropathy relies heavily on history and physical exam. Conservative treatment can be attempted, but if symptoms persist, surgical decompression is an effective alternative. Considering these findings, we argue that increased screening of at-risk patients aimed to recognize early signs of peroneal neuropathy might be useful to lighten the toll falls take on society.

## Supplementary Information

Below is the link to the electronic supplementary material.

Supplementary file1 Video 1. Clinical examination of common peroneal nerve compression, performed by Dr. PhD Elisabet Hagert. (MP4 59014 KB)
